# The Effect of Lipid Extract of *Nannochloropsis oceanica* Marine Microalgae on Glutathione and Thioredoxin-Dependent Antioxidant Systems in UVB-Irradiated Keratinocytes

**DOI:** 10.3390/md23120454

**Published:** 2025-11-26

**Authors:** Agnieszka Gęgotek, Maria Rosario Domingues, Pedro Domingues, Elżbieta Skrzydlewska

**Affiliations:** 1 Department of Analytical Chemistry, Medical University of Bialystok, Mickiewicza 2d, 15-222 Bialystok, Poland; elzbieta.skrzydlewska@umb.edu.pl; 2 Centre for Environmental and Marine Studies, Department of Chemistry, University of Aveiro, Santiago University Campus, 3810-193 Aveiro, Portugal; mrd@ua.pt; 3 Mass Spectrometry Centre, LAQV-REQUIMTE, Department of Chemistry, University of Aveiro, Santiago University Campus, 3810-193 Aveiro, Portugal; p.domingues@ua.pt

**Keywords:** lipid extract, marine microalgae, proteomics, keratinocytes, glutathione, thioredoxin

## Abstract

UVB radiation present in sunlight is the main pro-oxidative and pro-inflammatory factor that reaches human skin cells, including keratinocytes. Therefore, protective compounds eliminating the negative impact of UVB radiation are constantly being sought. This study aimed to estimate the effect of the lipid extract of microalgae *Nannochloropsis oceanica* (*N.o.*) on UVB-irradiated keratinocytes. A proteomic approach was used to estimate the proteomic profile of in vitro-treated keratinocytes. The results indicated 270 proteins had significantly altered expression in UVB-irradiated keratinocytes, while the treatment of cells with *N.o.* extract partially restored the levels of these proteins. Moreover, changes in protein structure resulting from the binding of glutathione (GSH) and thioredoxin (Trx) were also observed. Most of the GSH-modified proteins were involved in GSH or prostaglandin metabolism, while Trx-modified proteins were molecules related to Trx metabolism, as well as antioxidant and anti-inflammatory signaling. The treatment of cells with *N.o.* extract contributed to reversing the changes in the level of modification in individual proteins. It can be suggested that the lipid components of the microalgae *N.o.* extract protect keratinocytes against changes in metabolism induced by UVB radiation, modulating the antioxidant and pro-inflammatory responses of cells at the GSH and Trx-based signaling levels.

## 1. Introduction

Marine microalgae are among the most important components of the oceanic ecosystem, and, due to their rapid growth rates and ability to survive in harsh environments, they are increasingly used in food production. Moreover, the use of microalgae has been gradually increasing, including in the production of food, medicines, and cosmetics [1]. Many studies have shown that microalgae extracts have a variety of functions in regulating health/metabolism and preventing disease, owing to their antitumor, antiviral, antioxidant, anti-inflammatory, and antithrombotic properties [2]. These findings direct scientific research toward the identification of cytoprotective/medicinal compounds in lipid extracts from individual algal species. One of the very promising microalgae species for human health protection might be *Nannochloropsis oceanica* (*N.o.*). These marine green single-celled microalgae have so far been used to produce nutraceuticals and feed supplements [3]. Studies have shown that lipid extracts obtained from microalgae, such as *Nannochloropsis* sp., as well as others (including *Chlorella* sp. and *Chlorococcum* sp.), possess antioxidant, anti-inflammatory, and antibacterial properties [4]. This is related to their ability to suppress the production of pro-inflammatory cytokines, including tumor necrosis factor alpha (TNFα), interleukin 1β (IL-1β), and interleukin 6 (IL-6), and thus to reduce inflammation [5,6]. It has been shown that the lipid extract of *N.o.* is rich in long-chain omega-3 polyunsaturated fatty acids, but also phospholipids, glycolipids, and betaine lipids [7]. These molecules act as modulators of cellular metabolism and as cell protectors, especially in UV-irradiated skin cells such as skin fibroblasts and keratinocytes [8,9], as well as in UV-irradiated keratinocytes from psoriatic skin lesions [10].

The protective effect of *N.o.* lipid extract is explained by its influence on anti-inflammatory signaling via lipid mediators, such as eicosanoids and endocannabinoids [11]. On the other hand, these mediators significantly change the expression and activity of cytoprotective proteins, including enzymatic and nonenzymatic antioxidants [9]. In skin cells, it involves stimulating the activity of cytosolic and mitochondrial superoxide dismutases (SOD1 and SOD2) and catalase (CAT), which are decreased by cells’ exposure to UVB radiation [9]. Moreover, *N.o.* lipid extract also significantly induces glutathione (GSH) and thioredoxin (Trx)-dependent antioxidant systems, indicating a specific role for these systems in cell-supported protection against the harmful effects of UVB radiation on epidermal cells [9]. However, the GSH-dependent system (GSH, GSHPx, GSSGR), as well as the Trx-dependent system (Trx, TrxR), have for many years been identified as some of the basic antioxidant systems that combine the action of both antioxidant enzymes and nonenzymatic molecules in skin cells protection against UVA/B irradiation [12] and are important for the protection of the entire proteome exposed to oxidative modifications resulting from cell exposure to harmful radiation.

Therefore, the aim of this study was to assess the extent to which the lipid extract of *N.o.* prevents UVB-induced disturbances in the keratinocyte proteome. This involved an evaluation of the functions of proteins with altered expression, as well as an analysis of GSH- and Trx-protein adducts that influence the antioxidant defense of these skin cells.

## 2. Results

**Figure 1:**
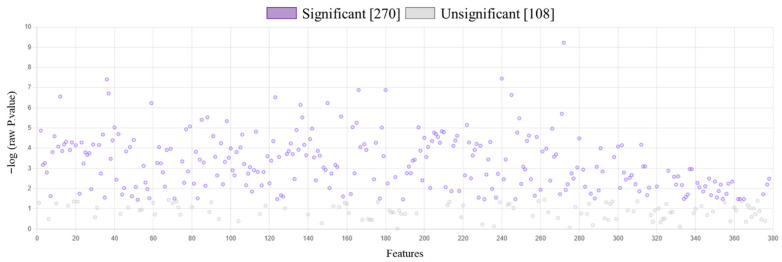
The results of ANOVA (FDR < 5%) for proteins expression identified in keratinocytes following UVB radiation [312 nm; 60 mJ/cm^2^] and/or treated with lipid extract of *Nannochloropsis oceanica* [3 µg/mL]. Each cell variant was repeated in 3 independent biological replicates. Of the 378 proteins, 270 were identified with statistically significantly changed expression, which represented over 70% of the quantified proteins. The list of proteins and their *p* value are presented in Supplementary Table S2.

**Figure 2:**
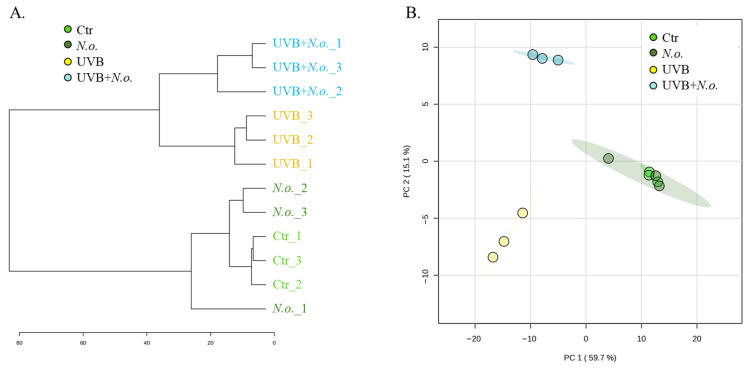
The dendrogram (**A**) and principal components (PC) analysis (**B**) of proteins expression identified in control keratinocytes (Ctr), as well as in these cells following UVB radiation [312 nm; 60 mJ/cm^2^] and/or treated with lipid extract of *Nannochloropsis oceanica* (*N.o.*) [3 µg/mL]. Each cell variant was repeated in 3 independent biological replicates. Both the dendrogram creation and PC analysis indicated a clear separation of the UVB-exposed cells from the other keratinocytes. At the same time, it was identified that the proteome of the Ctr and *N.o.* treated cells did not differ sufficiently to separate these groups.

Obtained proteomic data of keratinocytes exposed to UVB radiation and/or treated with the lipid extract of *N.o.* enabled us to identify and quantify 2235 proteins, of which 378 in each tested sample were identified with at least 2 unique peptides longer than 6 amino acid residues (Supplementary Table S1). The results of statistical analysis (ANOVA (FDR < 5%)) of the expression of identified proteins indicated 270 proteins with statistically significant changes in expression (Figure 1). These results enabled us to clearly differentiate between non-irradiated and UVB-irradiated keratinocytes. Both the dendrogram and principal components (PC) analysis separated keratinocytes exposed to UVB from non-irradiated ones and from cells treated with the lipid extract of *N.o.* (UVB + *N.o.*) following UVB irradiation. Only the differences between the control (Ctr) and extract-treated cells (*N.o.*) were small enough to prevent distinguishing these samples as autonomous lines in the dendrogram, or separating these groups in the PC plot (Figure 2).

**Figure 3:**
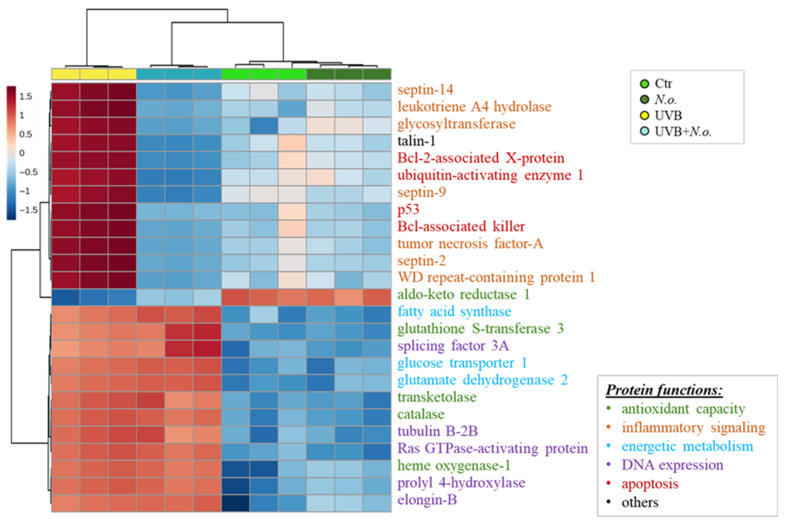
The heatmap of top 25 proteins that expression has been the most changed in control keratinocytes (Ctr), as well as in these cells following UVB radiation [312 nm; 60 mJ/cm^2^] and/or treated with lipid extract of *Nannochloropsis oceanica* (*N.o.*) [3 µg/mL]. Each cell variant was repeated in 3 independent biological replicates. The most changed by UVB radiation group of proteins are up-regulated proteins involved in pro-inflammatory signaling and the expression of these proteins were silenced by the treatment of cells with *N.o*. The other group up-regulated by UVB irradiation proteins were cytoprotective molecules, as well as proteins involved in energetic metabolism and DNA expression, that were partially also up regulated by algae extract treatment following irradiation.

**Figure 4:**
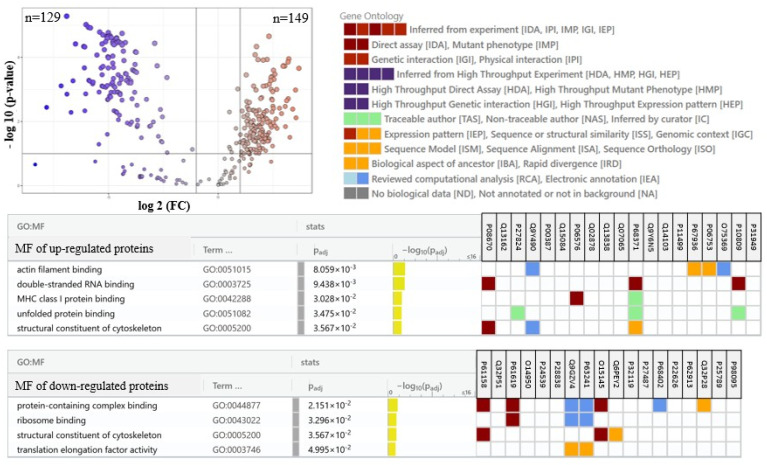
The volcano plot of proteins expression in keratinocytes following UVB radiation [312 nm; 60 mJ/cm^2^] comparing to control cells and the tables showing molecular function of the 20 top modified (up- or down-regulated) proteins. Lower number than 20 proteins shown in table resulting from the lack of indicated protein in the GO database. Each cell variant was repeated in 3 independent biological replicates. Individual comparison between UVB irradiated keratinocytes and control cells indicated 149 up-regulated and 129 down-regulated proteins with various molecular functions, including actin filament/RNA/ribosome/protein complex/cytoskeleton binding, as well as elongation factor activity. The *p*-values and the fold change for each protein are included in Supplementary Table S3. Abbreviations: FC, fold change; GO, gene ontology; MF, molecular function.

**Figure 5:**
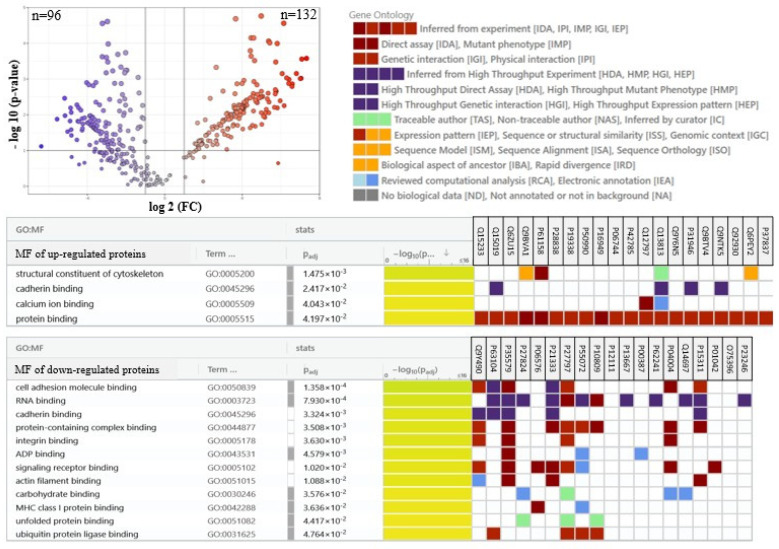
The volcano plot of proteins expression in keratinocytes following UVB radiation [312 nm; 60 mJ/cm^2^] comparing to these irradiated cells following their treatment with lipid extract of *Nannochloropsis oceanica* [3 µg/mL] and the tables showing molecular function of the 20 top modified (up- or down-regulated) proteins. Lower number than 20 proteins shown in table resulting from the lack of indicated protein in the GO database. Each cell variant was repeated in 3 independent biological replicates. Individual comparison between UVB irradiated keratinocytes and cells treated with algae lipid extract following UVB irradiation indicated 132 up-regulated and 96 down-regulated proteins with various molecular functions, including actin filament/RNA/protein/protein complex/calcium ion/signaling receptor/carbohydrate binding. The *p*-values and the fold change for each protein are included in Supplementary Table S4. Abbreviations: FC, fold change; GO, gene ontology; MF, molecular function.

The main factor responsible for such a clear separation between sample groups was changes in the expression of the top altered proteins, the most significant 25 of which were presented in the heatmap (Figure 3). These results show that the group of proteins most changed by UVB radiation are upregulated proteins involved in pro-inflammatory signaling (including septins 2, 9 and 14 (Q15019, Q9UHD8, Q6ZU15), leukotriene A4 hydrolase (P09960), glycosyltransferase (Q6Y288), tumor necrosis factor-A (P01375), WD repeat-containing protein 1 (O75083)), as well as apoptosis (Bcl-2-associated X-protein (Bax, Q07812), p53 (P04637), Bcl-associated killer (Bak, Q16611), ubiquitin-activating enzyme 1 (Q9GZZ9)), and the expression of these proteins were silenced by the treatment of cells with *N.o*. The other group of proteins upregulated by UVB irradiation were cytoprotective molecules, such as glutathione S-transferase 3 (Q16772), transketolase (P29401), catalase (P04040), as well as proteins involved in energetic metabolism (e.g., glutamate dehydrogenase 2 (P49448), glucose transporter 1 (P13866), fatty acid synthase (P49327)), and DNA expression (elongin-B (Q15370), prolyl 4-hydroxylase (P13674), Ras GTPase-activating protein (P20936), tubulin B-2B (Q9BVA1), splicing factor 3A (Q12874)) that were also partially upregulated by the algae extract treatment following irradiation. On the other hand, these cytoprotective proteins in non-irradiated keratinocytes were slightly downregulated by the algae extract (Figure 3). Among the top 25 modified proteins, only aldo-keto reductase 1 (C9JRZ8) was downregulated by UVB irradiation, and the algae extract partially countered this effect (Figure 3). An individual comparison between UVB-irradiated keratinocytes and control cells (Figure 4) revealed 149 upregulated and 129 downregulated proteins with various molecular functions, whereas treatment of cells with the algae lipid extract following UVB irradiation reduced these numbers to 132 and 96, respectively (Figure 5). Moreover, the functions of proteins with altered expression were changed more significantly than in experimental keratinocytes not treated with the extract (Figure 5).

**Figure 6:**
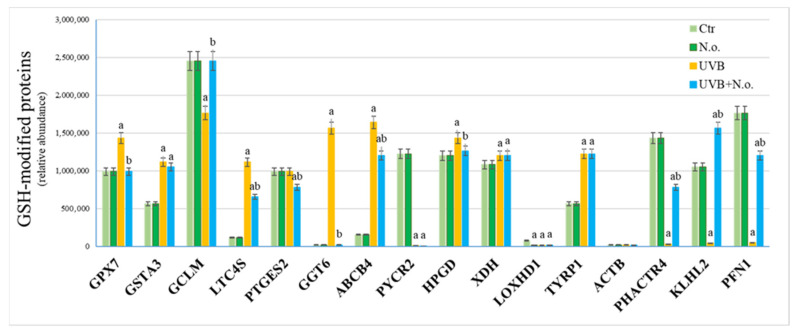
The level of proteins covalently modified by GSH in control keratinocytes (Ctr), as well as in these cells following UVB radiation [312 nm; 60 mJ/cm^2^] and/or treated with lipid extract of *Nannochloropsis oceanica* (*N.o*.) [3 µg/mL]. Each cell variant was repeated in 3 independent biological replicates. UVB radiation enhanced GSH binding to GPX7, GSTA3, LTC4S, GGT6, ABCB4, HPGD, XDH and TYRP1, simultaneously, reducing GSH-adducts formation with GCLM, PYCR2, PHACTR4, KLHL2 and PFN1. Algae extract treatment reduced the effect of UVB radiation by partially restoring the initial values of the levels of GSH adducts with proteins such as GPX7, GCLM, LTC4S, GGT6, ABCB4, HPGD, PHACTR4, KLHL2 and PFN1. Mean values ± SD are presented with statistically significant differences (*p* < 0.05) marked with ‘a’ or ‘b’ (comparing to Ctr or UVB irradiated group, respectively). Abbreviations: ABCB4, phosphatidylcholine translocator; ACTB, actin; GCLM, glutamate-cysteine ligase; GGT6, glutathione hydrolase 6; GPX7, glutathione peroxidase 7; GSH, glutathione; GSTA3, glutathione-S-transferase; HPGD, 15-hydroxyprostaglandin dehydrogenase [NAD(+)]; KLHL2, Kelch-like protein 2; LOXHD1, lipoxygenase; LTC4S, leukotriene C4 synthase; PFN1, profilin-1; PHACTR4, phosphatase and actin regulator 4; PTGES2, prostaglandin E synthase 2; PYCR2, pyrroline-5-carboxylate reductase 2; TYRP1, 5,6-dihydroxyindole-2-carboxylic acid oxidase; XDH, xanthine dehydrogenase/oxidase.

**Figure 7:**
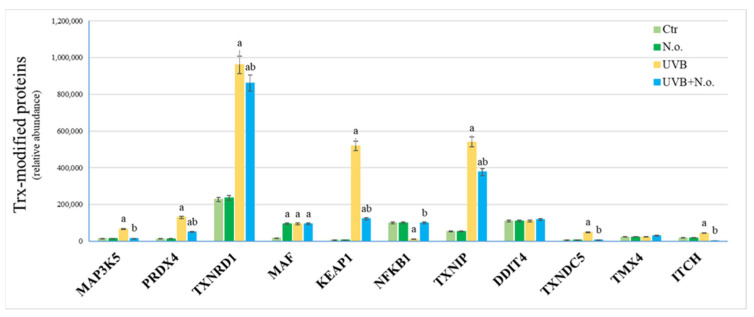
The level of proteins covalently modified by Trx in control keratinocytes (Ctr), as well as in these cells following UVB radiation [312 nm; 60 mJ/cm^2^] and/or treated with lipid extract of *Nannochloropsis oceanica* (*N.o.*) [3 µg/mL]. Each cell variant was repeated in 3 independent biological replicates. UVB radiation was the main factor significantly enhancing the level of Trx-modified proteins, such as MAP3K5, PRDX4, TXNRD1, MAF, KEAP1, TXNIP, TXNDC5 and ITCH. Only the level of Trx-modified NFKB1was decreased following keratinocytes exposure to UVB radiation comparing to the Ctr. In all these cases *N.o.* extract reduced the effect of UVB radiation by partially restoring the levels of Trx-modified proteins. Mean values ± SD are presented with statistically significant differences (*p* < 0.05) marked with ‘a’ or ‘b’ (comparing to Ctr or UVB irradiated group, respectively). Abbreviations: DDIT4, DNA damage-inducible transcript 4 protein; ITCH, E3 ubiquitin-protein ligase; KEAP1, Kelch-like ECH-associated protein 1; MAF, transcription factor Maf; MAP3K5, mitogen-activated protein kinase 5; NFKB1, nuclear factor NF-kappa-B (NFκB p105); PRDX4, peroxiredoxin-4; TMX4, thioredoxin-related transmembrane protein 4; Trx, thioredoxin; TXNDC5, thioredoxin domain-containing protein 5; TXNIP, thioredoxin-interacting protein; TXNRD1, thioredoxin reductase 1.

**Figure 8:**
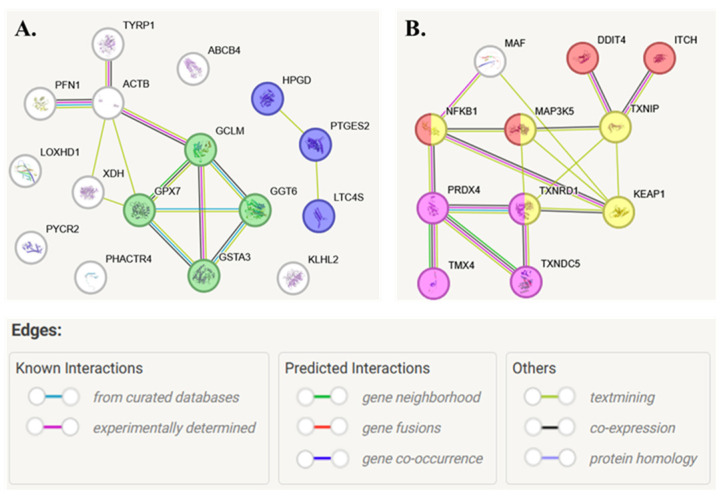
The protein-protein associations found for GSH (**A**) or Trx (**B**) modified molecules in keratinocytes following UVB radiation [312 nm; 60 mJ/cm^2^] and/or treated with lipid extract of *Nannochloropsis oceanica* [3 µg/mL]. Each cell variant was repeated in 3 independent biological replicates. Local network clusters indicated following pathways: green—glutathione metabolism; pink—thioredoxin metabolism; red—apoptosis; violet—prostaglandin metabolism; yellow—cellular responses to stress. Abbreviations: ABCB4, phosphatidylcholine translocator; ACTB, actin; DDIT4, DNA damage-inducible transcript 4 protein; GCLM, glutamate-cysteine ligase; GGT6, glutathione hydrolase 6; GPX7, glutathione peroxidase 7; GSH, glutathione; GSTA3, glutathione-S-transferase; HPGD, 15-hydroxyprostaglandin dehydrogenase [NAD(+)]; ITCH, E3 ubiquitin-protein ligase; KEAP1, Kelch-like ECH-associated protein 1; KLHL2, Kelch-like protein 2; LOXHD1, lipoxygenase; LTC4S, leukotriene C4 synthase; MAF, transcription factor Maf; MAP3K5, mitogen-activated protein kinase 5; NFKB1, nuclear factor NF-kappa-B (NFκB p105); PFN1, profilin-1; PHACTR4, phosphatase and actin regulator 4; PRDX4, peroxiredoxin-4; PTGES2, prostaglandin E synthase 2; PYCR2, pyrroline-5-carboxylate reductase 2; TMX4, thioredoxin-related transmembrane protein 4; Trx, thioredoxin; TXNDC5, thioredoxin domain-containing protein 5; TXNIP, thioredoxin-interacting protein; TXNRD1, thioredoxin reductase 1; TYRP1, 5,6-dihydroxyindole-2-carboxylic acid oxidase; XDH, xanthine dehydrogenase/oxidase.

Immunoprecipitation of proteins covalently bound to GSH or Trx indicated that both UVB radiation and *N.o.* lipid extract significantly influenced the level of modified proteins (Figure 6 and Figure 7). In the case of GSH, UVB radiation enhanced its binding to glutathione peroxidase 7 (GPX7, Q96SL4), glutathione-S-transferase (GSTA3, Q16772), leukotriene C4 synthase (LTC4S, Q16873), glutathione hydrolase 6 (GGT6, Q6P531), phosphatidylcholine translocator (ABCB4, P21439), 15-hydroxyprostaglandin dehydrogenase [NAD(+)] (HPGD, P15428), xanthine dehydrogenase/oxidase (XDH, P47989), and 5,6-dihydroxyindole-2-carboxylic acid oxidase (TYRP1, P17643) (Figure 6). Simultaneously, UVB reduced GSH-adducts formation with glutamate-cysteine ligase (GCLM, P48507), pyrroline-5-carboxylate reductase 2 (PYCR2, Q96C36), phosphatase and actin regulator 4 (PHACTR4, Q8IZ21), Kelch-like protein 2 (KLHL2, O95198), and profilin-1 (PFN1, P07737). Moreover, the algae extract treatment reduced the effect of UVB radiation by partially restoring the initial values of the levels of GSH adducts with proteins such as GPX7, GCLM, LTC4S, GGT6, ABCB4, HPGD, PHACTR4, KLHL2, and PFN1 (Figure 6). According to local network clustering (based on the Gene Ontology database), half of these proteins are involved in dual pathways: glutathione or prostaglandin metabolism (Figure 8A).

In the case of Trx-modified proteins UVB radiation was the main factor significantly enhancing the level of modified proteins, such as mitogen-activated protein kinase 5 (MAP3K5, ASK1, Q99683), peroxiredoxin-4 (PRDX4, Q13162), thioredoxin reductase 1 (TXNRD1, Q16881), transcription factor Maf (MAF, O75444), Kelch-like ECH-associated protein 1 (KEAP1, Q14145), thioredoxin-interacting protein (TXNIP, Q9H3M7), thioredoxin domain-containing protein 5 (TXNDC5, Q8NBS9), and E3 ubiquitin-protein ligase (ITCH, Q96J02). Only the level of Trx-modified nuclear factor NF-kappa-B (NFKB1, NFκB p105, P19838) decreased following keratinocytes’ exposure to UVB radiation compared to the control cells. In all these cases, *N.o.* extract treatment reduced the effect of UVB radiation by partially restoring the levels of Trx-modified proteins (Figure 7). Most of the Trx-modified proteins are involved in thioredoxin metabolism and in intracellular signaling pathways responsible for responses to stress or apoptosis (Figure 8B).

## 3. Discussion

UV radiation is one of the most dangerous and at the same time the most common physical environmental factors affecting the functioning of human skin cells. The outermost layer of the skin, the epidermis, is mainly created by the keratinocytes that are most severely affected by the high-energy UVB radiation [13]. Results from large-scale proteomic, lipidomic, and interdisciplinary studies show that UVB radiation is highly destructive to keratinocyte metabolism [14,15]. Therefore, there is a constant search for natural compounds that have the least possible impact on cellular metabolism and simultaneously protect cells from the harmful effects of UVB radiation.

The growing interest in the design of skin protection/care products has led to extracts from sea algae, including microalgae *Porphyridium cruentum*, *Phaeodactylum tricornutum*, and *Nannochloropsis gaditana*, being widely used in cosmetics due to their antioxidant properties [16]. Microalgae extracts are rich in antioxidants, vitamins, and essential fatty acids, which have proven their importance for skin health, promoting hydration, rejuvenation, and protection from environmental stress. Moreover, microalgae produce bioactive compounds with anti-inflammatory properties, which can be useful in treatments for skin diseases, as in the case of *Cyanobacterium aponinum* or *Spirulina* sp., whose extracts are used in acne therapy [17]. Moreover, the lipophilic nature of the epidermal intercellular matrix (rich in keratin, ceramides, and other lipid derivatives) favors the use of lipid extracts from these microorganisms [18].

The lipid extract of *Nannochloropsis oceanica* algae used in this experiment has already been tested for skin cell protection against the degenerative effects of UVA/UVB [8,9,19,20], as well as against the development of pro-inflammatory skin diseases such as psoriasis [10], or even as a potential support in anti-melanoma therapy [21]. Moreover, results from this study confirm previous observations by significantly silencing the expression of pro-inflammatory and pro-apoptotic proteins in keratinocytes after exposure to UVB radiation, while simultaneously increasing the expression of antioxidant proteins and proteins involved in energy metabolism. Moreover, previous studies indicated that, although the extract did not significantly affect the levels of GSH or Trx in keratinocytes, antioxidant systems dependent on these small molecules were activated by *N.o.* extract following the exposure of cells to UVB [9]. These results appear meaningful, as the microalgae extract also restores keratinocyte viability reduced by UVB radiation [9].

GSH is a common antioxidant consisting of three amino acids, glutamic acid, glycine, and cysteine. Consequently, reduced GSH can act as a proton donor or trap electrophiles. Therefore, GSH is known as a direct scavenger of free radicals and as a molecule that can participate in the reduction of oxidized proteins, e.g., as a cofactor of glutathione peroxidase [22]. So far, GSH adduct formation with proteins occurs via interaction with cysteine (via Michael addition) [23]. The properties of GSH to bind proteins are based on the multidirectional action of this molecule. In the case of enzymes involved in its metabolism, GSH binding favors enzyme activity, which can be applied for GCLM and GGT6 (enzymes involved in GSH biosynthesis/degradation) [24], but also GPX7 and GSTA3, which use GSH to detoxify hydrogen peroxide and lipid hydroperoxides, as well as harmful xenobiotics [25]. UVB reduces GSH-GCLM adducts and increases GSH-GGT6, thereby blocking the action of the GSH-dependent antioxidant system at the stage of GSH biosynthesis. The microalgae extract prevents only UVB-induced changes caused at the level of GSH-GCLM adducts, thereby unblocking the first step of GSH biosynthesis, but there is no visible effect on the level of its degradation catalyzed by GGT6. At the same time, the microalgae extract completely prevents UVB-induced formation of GSH adducts with GPX7 and GSTA3, suggesting decreased activity of these enzymes. However, this may be due to the antioxidant effect of the extract itself, so there is no need for the cell to activate this system. On the other hand, GSH is also bonded to the enzymes involved in prostaglandin metabolism, including HPGD, LOXHD1, LTC4S, and PTGES2. HPGD is an oxidoreductase that generates 15-keto-prostaglandin E2, a molecule that, by activating EP2 and EP4 receptors, stimulates pro-inflammatory signaling [26]. LOXHD1 is a catalyst of the dioxygenation of polyunsaturated fatty acids with the generation of eicosanoids [27], while LTC4S and PTGES2 are responsible for leukotriene C4 and prostaglandin E biosynthesis, respectively [28]. All products of the activity of the mentioned enzymes are involved in pro-inflammatory signaling and are known to contribute to inflammatory diseases. So far, there is no data on the effect of GSH attachment to these enzymes, whether it increases or inhibits their activity. Therefore, it can only be suggested that the observed increase in GSH-LTC4S and GSH-HPGD adducts generation after keratinocytes’ exposure to UVB, observed in this study, is related to pro-inflammatory signaling associated with the increase in 15-keto-prostaglandin E2 or leukotriene C4 levels in UV irradiated various epithelial cell lines [29,30]. Moreover, treatment of UVB-irradiated cells with a microalgae lipid extract significantly reduces the levels of GSH adducts with all of the above-mentioned enzymes involved in prostaglandin metabolism, demonstrating that this extract influences prostaglandin-based signaling under conditions of oxidative stress. This statement is supported by the anti-inflammatory effect of the used extract, including silencing NFKB-based signaling [9]. Nevertheless, GSH-protein adduct formation is often associated with the detoxification of damaged or destined-for-removal proteins [31], and the levels of which in UVB-irradiated keratinocytes are significantly higher than in unexposed cells [14]. Therefore, the reversal of this effect by algae extract after cells are exposed to UVB indicates its cytoprotective capacity, which may occur at the stage of protein marking for elimination or even prevent their damage.

At the same time, UVB radiation favors the formation of protein adducts with Trx. The level of free Trx in UVB-irradiated keratinocytes is reduced by half compared to unirradiated cells [32], which may suggest the consumption of this molecule by the cell as a result of eliminating the pro-oxidant effect of UVB radiation. It is accompanied by a decrease in the activity of Trx-dependent antioxidant enzymes, such as TXNRD [33]. On the other hand, the activity of the Trx-based antioxidant system might be stimulated by the Trx binding to enzymes of which it is a cofactor, including TXNRD, but also TXNDC5 and TMX4, that are modified by Trx in keratinocytes following UVB irradiation. Lipid algal extract reduces the levels of these modifications, thereby restoring the free Trx pool in the cytoplasm [9]. However, it is not known how this affects the activity of Trx-dependent enzymes. Moreover, TXNIP, also known as Vitamin-D upregulated protein-1 (VDUP-1), interacts with thioredoxin to regulate redox responses, and has been identified as a factor determining the pathogenesis of skin diseases (e.g., psoriasis) during keratinocyte differentiation [34].

The proteins found to be significantly modified by Trx have also been clustered into molecules involved in cellular responses to oxidative stress and apoptosis (Figure 8B). This is particularly evident in the case of NFKB, a transcription factor that regulates the expression of pro-inflammatory signaling proteins [35]. Its activity is regulated by forming a complex with IKB. NFKB is dissociated from the complex after directed phosphorylation of IKB, usually catalyzed by a dedicated IKK kinase [36]. However, Trx might bind to NFKB, and it is assumed that the formation of these adducts prevents the activation of NFKB1 even after its dissociation from IKB by blocking NFKB translocation to the nucleus [37,38]. Moreover, it has been shown that NFKB conjugated to a thiol molecule (e.g., Trx) is degraded and removed from the cell [39]. Additionally, the exposure of cells to UVB radiation prevents the formation of such adducts, thereby facilitating the induction of NFKB-based pro-inflammatory signaling in UVB-irradiated keratinocytes [40]. Simultaneously, Trx might also bind to KEAP1, a cytosolic inhibitor of Nrf2 that induces the expression of cytoprotective proteins. By binding Nrf2, KEAP1 inhibits its transcriptional activity. On the other hand, changes in KEAP1 conformation allow Nrf2 to dissociate and be activated, which occurs mainly under pro-oxidant conditions [41]. Following exposure of keratinocytes to UVB radiation, KEAP1 is extensively modified by Trx, altering its structure and preventing Nrf2 binding. As a result, under pro-oxidative conditions induced by UVB radiation, increased Nrf2 transcriptional activity is noted in skin cells [42,43]. As a result, both UVB-induced Trx-KEAP1 complex, as well as reduced Trx-NFKB1 adduct levels, induce a protective antioxidant response against pro-inflammatory signaling in keratinocytes. On the other hand, treating keratinocytes with lipid algae extract without UV radiation does not significantly affect the levels of these adducts; however, following UVB irradiation, *N.o.* significantly reduces Trx-KEAP1 and enhances Trx-NFKB1 adduct levels, thereby counteracting the cellular effects of UVB radiation.

In conclusion, lipid components of the microalgae *Nannochloropsis oceanica* extract protect keratinocytes against UVB-induced changes in protein expression, without altering the proteome of unexposed cells. The applied lipid extract prevents UVB-induced changes in keratinocyte protein expression and prevents modifications by GSH or Trx, suggesting its role in modulating the antioxidant and pro-inflammatory responses of cells to UVB-induced stress. The observed effect of the microalgae *N.o.* may serve as a basis for using this extract to protect the skin against harmful environmental factors.

## 4. Materials and Methods

### 4.1. Microalgae Lipid Extracts

The microalgae *Nannochloropsis oceanica* (*N.o.*) were cultivated in Guillard’s F2 culture medium adapted to local water as described previously [9,44]. Microalgae biomass was spray-dried and supplied by Allmicroalgae, Natural Products S.A., located in Rua 25 de Abril s/n 2445-413 Pataias, Portugal.

Lipid extraction was performed using a modified Folch method [7,45]. Briefly, lipids were extracted using a solvent mixture of dichloromethane: methanol (2:1, *v*/*v*), which was added to 25 mg of biomass. The samples were vortexed and centrifuged at 670× *g* for 10 min. The supernatant was then collected, and this process was repeated three more times. The combined supernatants were dried under a stream of nitrogen. The extracts were then redissolved in dichloromethane and methanol and vortexed thoroughly. Mili-Q water was subsequently added. Phase separation was achieved after centrifugation (670× *g* for 10 min), and the organic phase was collected. The aqueous phase was re-extracted twice more. The lipid extract was obtained by combining the organic phases. The lipid profile of the obtained *Nannochloropsis oceanica* extract was characterized by hydrophilic interaction liquid chromatography coupled with high-resolution mass spectrometry (HILIC–MS) and tandem MS (MS/MS) using a Q-Exactive hybrid quadrupole Orbitrap mass spectrometer (Thermo Fisher Scientific, Bremen, Germany) as reported previously [7] (Supplementary Table S5).

### 4.2. Cell Culture and Treatment

Human immortalized keratinocytes CDD 1102 KERTr (CRL2310), obtained from the American Type Culture Collection ATCC^®^ (Manassas, VA, USA), were used in the experiments. Cells from passage 8 were cultured in a humid atmosphere of 5% CO_2_ and a temperature of 37 °C. The growth medium was prepared as follows: keratinocyte–SFM medium supplemented with 1% bovine pituitary extract (BPE) and antibiotics: 50 U/mL penicillin and 50 µg/mL streptomycin.

After reaching 70% confluency, the keratinocytes were exposed to UVB radiation. The radiation dose was 60 mJ/cm^2^ (312 nm, power density at 4.08 mW/cm^2^) (Bio-Link Crosslinker BLX 312, Vilber Lourmat, Eberhardzell, Germany), which corresponded to approximately 70% of cell viability as measured by the MTT (3-(4,5-dimethylthiazol-2-yl)-2,5-diphenyltetrazolium bromide) method [46].

To examine the effect of lipid extract from *N.o.*, following irradiation, cells were incubated for 24 h in medium supplemented with the extract at a concentration of 3 µg/mL, primarily dissolved in DMSO (dimethyl sulfoxide), with a final solution concentration of 0.1%. The concentration of the extract was selected based on previous studies [11], which showed that this extract had a protective effect on keratinocytes exposed to UVB radiation but did not alter the morphology or viability of unexposed cells. Control cells were cultured in parallel in medium containing 0.1% DMSO. Cell viability curves prepared to select the UVB dose and lipid extract concentration are presented in Supplementary File S1.

After 24 h of incubation, all cells were collected by scraping in cold PBS and disrupted by sonication on ice. Following centrifugation, the total protein content of the cell lysate was measured using the Bradford assay [47].

### 4.3. Isolation of GSH and Trx-Protein Adducts

Samples containing 100 µg proteins were pre-cleaned with protein A-agarose to remove molecules that could nonspecifically react with this substance. Protein A-agarose was removed by 1 min centrifugation (10,000× *g*, 4 °C). Next, the primary antibody against GSH or Trx (1:1000; Sigma-Aldrich; St. Louis, MO, USA) was added, and samples were incubated for 1 h at 4 °C. To precipitate the proteins bound with antibodies, protein A-agarose was added and incubated overnight. Subsequently, samples were centrifuged (10 min, 10,000× *g*, 4 °C), and the pellet obtained was received as proteins immunoprecipitated with GSH or Trx.

### 4.4. In-Solution Protein Digestion and Peptide Analysis

Cell lysates containing 50 µg of protein or immunoprecipitation adducts were mixed with 8 M urea for denaturation. Next, proteins were reduced with 10 mM 1,4-dithiothreitol and alkylated with 50 mM iodoacetamide. To stop alkylation, 10 mM 1,4-dithiothreitol was used again. Samples were diluted fourfold with ammonium bicarbonate buffer (AMBIC, 25 mM) and incubated for 17 h with sequencing-grade modified trypsin (Promega, Madison, WI, USA) at 37 °C. To stop the reaction, 10% formic acid was added to the samples, bringing the final concentration to 0.1% [48]. The obtained peptide mixture was dried under an inert gas.

Peptides obtained after digestion were dissolved in 5% acetonitrile with 0.1% formic acid and separated using a high-performance liquid chromatography system Ultimate 3000 (Dionex, Idstein, Germany) with a 50 mm × 75 µm PepMap RSLC capillary analytical C18 column (Dionex LC Packings, Dionex, Idstein, Germany) at a flow rate of 0.300 µL/min. Eluted peptides were analyzed using a Q Exactive HF mass spectrometer (Thermo Fisher Scientific, Bremen, Germany). The mass spectrometer was operated in a positive mode. Survey MS scans were conducted in the 200–2000 *m*/*z* range. The conditions of the peptide analysis were described in detail previously [49]. Tandem mass spectra were collected by the Xcalibur 4.1 software (Thermo Fisher Scientific, Bremen, Germany). Differences between samples in the intensity of randomly selected peaks were validated using the Agilent 1290 Infinity II LC system coupled to the Agilent 6475 triple quadrupole LC/MS system (Agilent Technologies, Heilbronn, Germany).

### 4.5. Protein Identification and Label-Free Quantification

Raw data were analyzed using freeware software MaxQuant v2.4.2 [50]. Input data were searched against the UniProtKB-SwissProt database (taxonomy: Homo sapiens, release September 2023). The default parameters were used for protein identification. Protein label-free quantification was performed according to the signal intensities of the precursor ions. The modified protein quantification was performed based on the peak area analysis. Only proteins with at least three peptides longer than 6 amino acid residues and at least two unique peptides were accepted for statistical analysis.

### 4.6. Statistical Analysis

Each cell variant was repeated 3 times in independent biological replicates. The results from individual protein label-free quantification were log-transformed, Pareto-scaled (mean-centered and divided by the square root of the standard deviation of each variable), and normalized by the sum of the protein intensities obtained for each sample using open-source software MetaboAnalyst 6.0 (http://www.metaboanalyst.ca (accessed on 11 January 2025)) [51]. The same software was used for biostatistical analysis, including univariate analysis (one-way ANOVA, Fisher’s least significant difference (LSD), false discovery rate (FDR) < 5%; multiple comparisons were corrected using the Benjamini–Hochberg procedure), volcano plots, heatmaps, and dendrogram creation. Protein–protein interactions were performed using the free tool STRING v11.5, based on the Gene Ontology (GO) database [52]. The functional enrichment analysis was performed using the g:Profiler (version e113_eg59_p19_f6a03c19) [53].

## Data Availability

The authors confirm that the data supporting the findings of this study are available within the article and its Supplementary Materials.

## Supplementary Materials

The following supporting information can be downloaded at: https://www.mdpi.com/article/10.3390/md23120454/s1, Supplementary Table S1. The IDs of the proteins that were identified and semi-quantified in control keratinocytes (Ctr), as well as in these cells following UVB radiation [312 nm; 60 mJ/cm^2^] and/or treated with lipid extract of *Nannochloropsis oceanica* (Algae) [3 µg/mL]. Data obtained using a high-performance liquid chromatography system Ultimate 3000 (Dionex, Idstein, Germany) coupled to a Q Exactive HF mass spectrometer (Thermo Fisher Scientific, Bremen, Germany). Raw data were analyzed by the MaxQuant v2.4.2 using the UniProtKB-SwissProt database (taxonomy: *Homo sapiens*, release 2024-09). Supplementary Table S2. The list of proteins that are expressed in keratinocytes following UVB irradiation [312 nm; 60 mJ/cm^2^] and/or treatment with lipid extract of *Nannochloropsis oceanica* (Algae) [3 µg/mL] was significantly changed (ANOVA, Fisher’s least significant difference (LSD), the false discovery rate (FDR) < 5%). Supplementary Table S3. The list of ID, *p*-values, and the fold changes (FC) of proteins with significantly changed expression in UVB [312 nm; 60 mJ/cm^2^] irradiated keratinocytes compared to control cells. Supplementary Table S4. The list of ID, *p*-values, and the fold changes (FC) of proteins with significantly changed expression in UVB [312 nm; 60 mJ/cm^2^] irradiated keratinocytes compared to these cells following their treatment with lipid extract of *Nannochloropsis oceanica* [3 µg/mL]. Supplementary Table S5. The lipid profile of the *Nannochloropsis oceanica* extract was characterized by hydrophilic interaction liquid chromatography coupled with high-resolution mass spectrometry (HILIC–MS) and tandem MS (MS/MS) using a Q Exactive hybrid quadrupole Orbitrap mass spectrometer (Thermo Fisher Scientific, Bremen, Germany). Supplementary File S1. The results of the MTT assay for keratinocytes treated with various doses of UVB radiation and concentrations of the lipid extract of *Nannochloropsis oceanica*.
