# Supplementary material for: The Effect of Lipid Extract of Nannochloropsis oceanica Marine Microalgae on Glutathione and Thioredoxin-Dependent Antioxidant Systems in UVB-Irradiated Keratinocytes
Source: Mar Drugs. 2025 Nov 26;23(12):454. doi: 10.3390/md23120454 (PMC12735059; doi:10.3390/md23120454)

Supplementary File S1. The results of the MTT assay for keratinocytes treated with various doses of UVB radiation and concentrations of the lipid extract of *Nannochloropsis oceanica*.

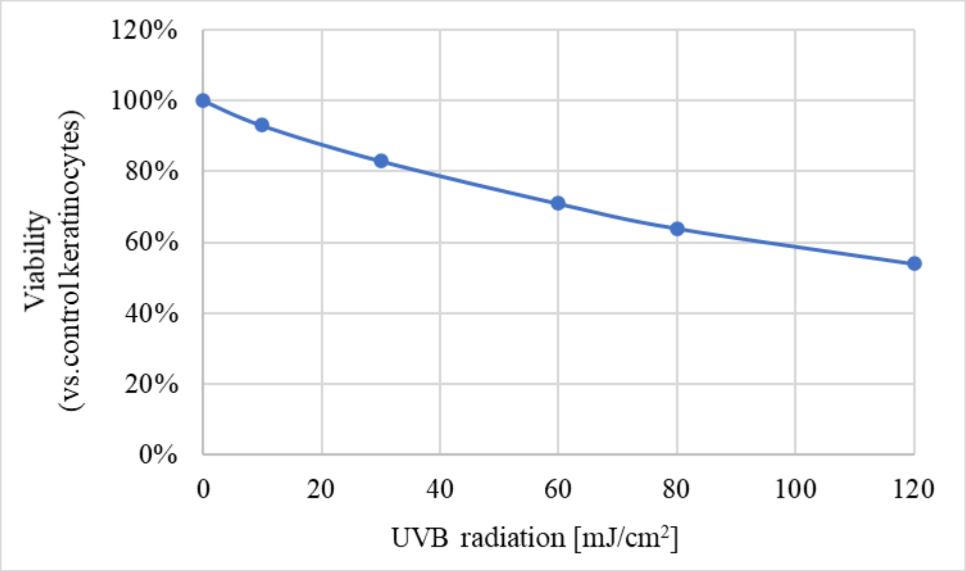

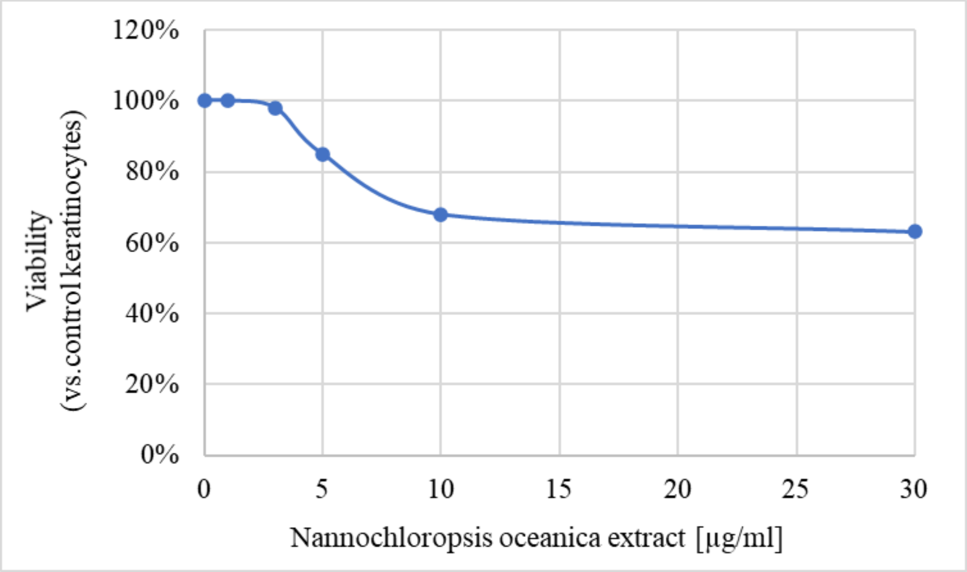

Supplement: Supplementary file 1 [file marinedrugs-23-00454-s001.zip › Supplementary file S1.pdf]
